# An update of the diagnosis, treatment, and prevention of leprosy: A narrative review

**DOI:** 10.1097/MD.0000000000039006

**Published:** 2024-08-23

**Authors:** Chien-Yuan Huang, Shih-Bin Su, Kow-Tong Chen

**Affiliations:** a Division of Occupational Medicine, Chi-Mei Medical Center, Liouying, Tainan, Taiwan; b Department of Occupational Medicine, Chi-Mei Medical Center, Tainan, Taiwan; c Department of Occupational Medicine, Tainan Municipal Hospital (managed by Show Chwan Medical Care Corporation), Tainan, Taiwan; d Department of Public Health, College of Medicine, National Cheng Kung University, Tainan, Taiwan.

**Keywords:** diagnosis, Hansen’s disease, leprosy, management, treatment

## Abstract

Leprosy is an infectious disease that remains a public health concern. It is caused by acid-fast Bacillus *leprae*, which primarily affects the skin and peripheral nerves, potentially leading to long-term disability and stigma. However, current and previous efforts have focused on developing better diagnostic and therapeutic interventions for leprosy, and its prevention needs to be addressed. In this review, we organize the currently published papers and provide updates on the global epidemiology, diagnosis, treatment, and prevention of leprosy. Several online databases, including MEDLINE (National Library of Medicine, Bethesda, MD), PubMed, EMBASE, Web of Science, and Google Scholar, were searched to collect relevant published papers. As a public health issue, the World Health Organization set the goal of leprosy elimination with a prevalence of <1 case per 10,000 people, which was achieved in 2000 and in most countries by 2010, mainly owing to the treatment of leprosy using drugs starting in 1980 and no-cost access for patients since 1995. Although diagnostic and therapeutic techniques have improved, the new occurrence of leprosy remains a critical global disease burden. With continuous technological improvements in diagnosing and treating leprosy, obtaining more relevant healthcare knowledge and preventing leprosy disability are crucial.

## 1. Introduction

Leprosy, also known as Hansen’s disease, is a chronic infectious disease caused by *Mycobacterium leprae*, or less commonly by *Mycobacterium lepromatosis*^[1]^ both of which cause similar pathological changes. *M leprae* belongs to the family *Mycobacteriaceae* and genus *Mycobacterium*.^[2]^ It preferentially invades peripheral nerve dermal histocytes and Schwann cells.^[2,3]^ *M leprae* is slow growing (approximately 12–14 days) and can survive outside the human body for 46 days.^[4,5]^ The incubation period for leprosy is long and broad, with 2 to 20 years estimates.^[5]^ *M leprae* prefers to grow at lower temperatures (27–33 °C); thus, it predominantly affects areas in the human body at lower temperatures.^[4,5]^

Dapsone monotherapy was initiated in the 1940s and was found to be effective; however, widespread resistance to dapsone was reported in the 1960s.^[6]^ The World Health Organization (WHO) recommended that all patients with leprosy should be treated with multidrug therapy (MDT) in 1981^[6]^ and its widespread execution in the 1990s to eliminate leprosy as a public health problem.^[7]^ In 1990, the WHO proposed eliminating leprosy globally by the end of the 20th century. However, the spread of the infection continued. In 2016, the WHO renewed the work “The Global Leprosy Strategy 2016 to 2020: Accelerating towards a leprosy-free world” to encourage all countries to enact specific strategies against stigma towards leprosy and decrease the incidence of leprosy with grade 2 disability (G2D) to less than 1 case per million.^[8]^ As leprosy remains a public health problem in many resource-limited countries despite many efforts, ensuring equality in access to diagnostic and rehabilitation facilities, treatment, and prevention of disability is crucial.^[9,10]^ Early detection and immediate and adequate treatment are essential strategies to prevent disease spread, physical disabilities, and deformities and to improve patients’ social lives.^[11–15]^ This study aims to provide updates on the changes in the diagnosis, treatment, and prevention of leprosy.

## 2. Methods

Different databases were used to tailor the search strategies. Several online databases, including MEDLINE (National Library of Medicine, Bethesda, MD), PubMed, EMBASE, Web of Science, and Google Scholar, were searched to select relevant published literature using a combination of the following keywords: “leprosy,” “Hansen’s disease,” “epidemiology,” “diagnosis,” “treatment,” and “prevention.” Only articles published in English were included in this study. Additional information was extracted from the reference lists of relevant papers. Articles not written in English, with no abstracts to the editor, or opinion articles were excluded from the review. Review articles were used as references to search for relevant studies.

## 3. Epidemiology

**Figure 1.**
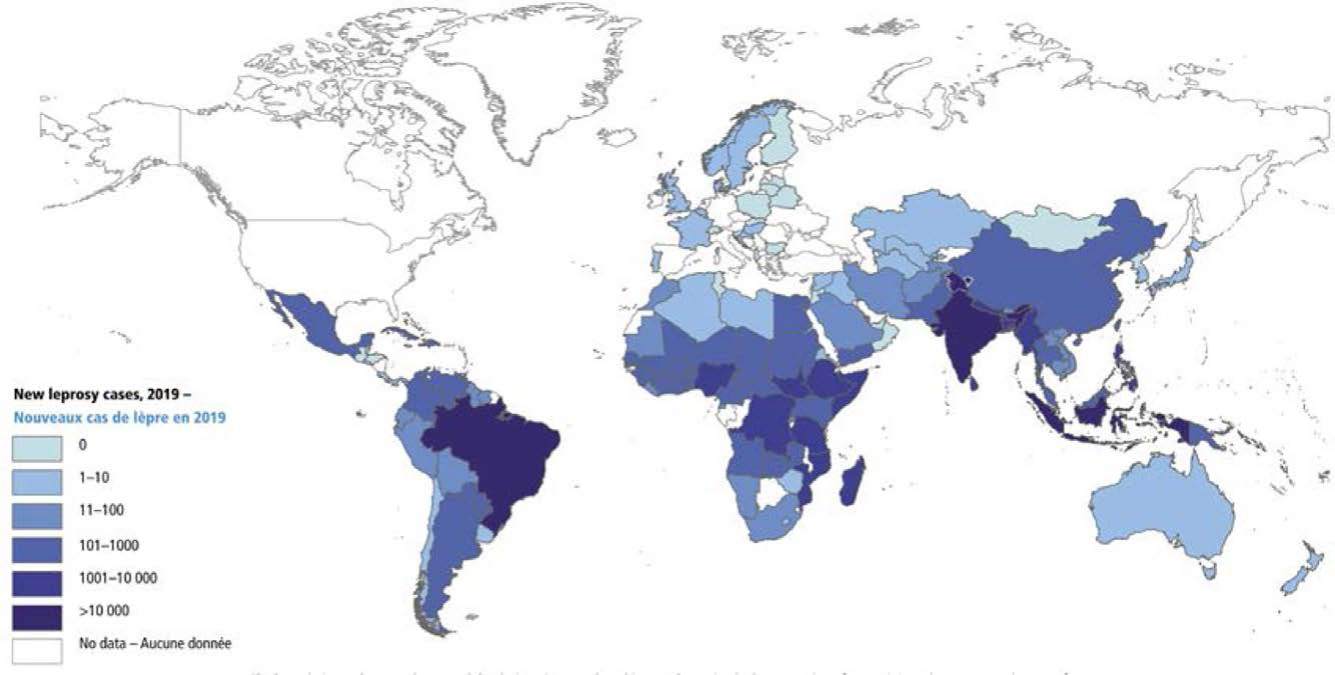
Global distribution of new leprosy cases in 2019 reported by World Health Organization (photograph courtesy of World Health Organization).

The introduction of MDT or polychemotherapy, as recommended by the WHO in 1981, has significantly reduced the global prevalence of leprosy cases.^[6,7]^ However, despite the widespread application of this therapy, 208,619 new leprosy cases were reported globally in 2018, only slightly reducing the number of new leprosy cases in 2019 reported by the WHO.^[15]^ Figure 1 is the map that reveals the distribution of new leprosy cases worldwide in 2019,^[15]^ with 202,185 incident cases reported globally. Countries in the African and Southeast Asian regions (SEAR) had the highest new case detection rates. Among these 143 countries, India (cases: 114,451), Brazil (27,863), and Indonesia (17,439) have the persistent presence of the most significant number of incident cases (>10,000). Meanwhile, 71% (10,661/14,981) of all new cases in children occurred in SEAR. A total of 10,813 new leprosy cases with G2D have been reported globally, with the highest number of G2D cases reported in Africa (2932), followed by America (2544), SEAR (817), and the West Pacific (262).^[15]^ Previous studies^[15,16]^ showed that the global pooled proportions of male and female patients with leprosy, multibacillary and paucibacillary leprosy, children and adult patients with leprosy, and G2D were 63% (95% confidence interval [CI]: 59–66%), 37% (95% CI: 34–41%), 69% (95% CI: 62–76%), 31% (95% CI: 24–38%), 11% (95% CI: 8–13%), 89% (95% CI: 87–92%), and 22% (95% CI: 15–30%), respectively. Hence, aside from the elaboration of effective drug treatment, more education and increasing accessibility to care are also important factors influencing the effectiveness of leprosy control.^[17–19]^

**Figure 2.**
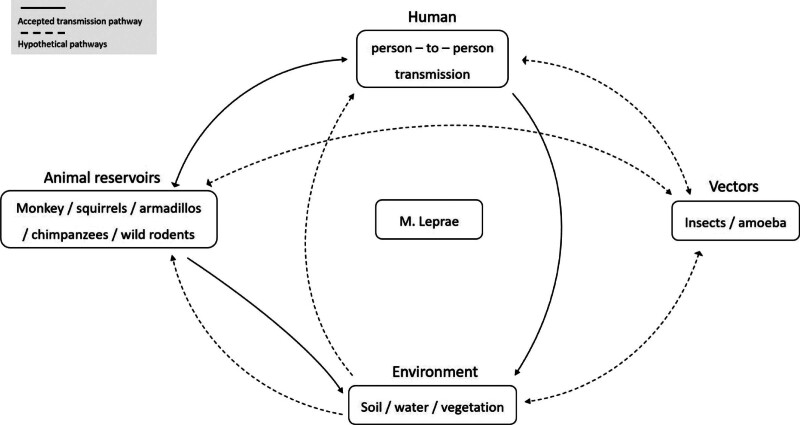
Potential transmission routes of leprosy.

Figure 2 shows the potential transmission routes of leprosy.^[1,20–25]^ Due to variability in host susceptibility to infection and a very long potential incubation period, the mechanism of transmission routes of *M leprae* still needs to be explored further; nevertheless, increased evidence of close and long-term living with infected patients and inhalation of bacilli through nasal secretions or respiratory droplets (aerosols) released by coughing and sneezing are suggested to be the main transmission routes.^[22–24]^ Humans are primary carriers of *M leprae*.^[26,27]^ However, in the Americas, *M leprae* has been cultivated in the footpads of mice, and previous studies have reported that it occurs naturally in wild armadillos, sooty mangabey monkeys, cynomolgus macaques, chimpanzees, and wild rodents.^[16,22]^ Hence, anthroponotic and zoonotic transmissions have been proposed as modes of transmission.^[20]^ Other potential transmission routes include blood, breast milk, insect bites, vertical transmission, handling, and blood transfusions^.[1,25]^

The main risk factors for *M leprae* infection include general clinical or social factors such as age, sex, close exposure to individuals with untreated leprosy, exposure to armadillos, immunosuppression, organ transplantation, chemotherapy, standard of living, spatial distribution, and genetic variation of the host.^[28,29]^ In non-endemic countries, leprosy cases are frequently observed in individuals who have lived in or spent a significant period in an endemic country or migrated from endemic regions.^[30]^

## 4. Clinical classification

**Figure 3.**
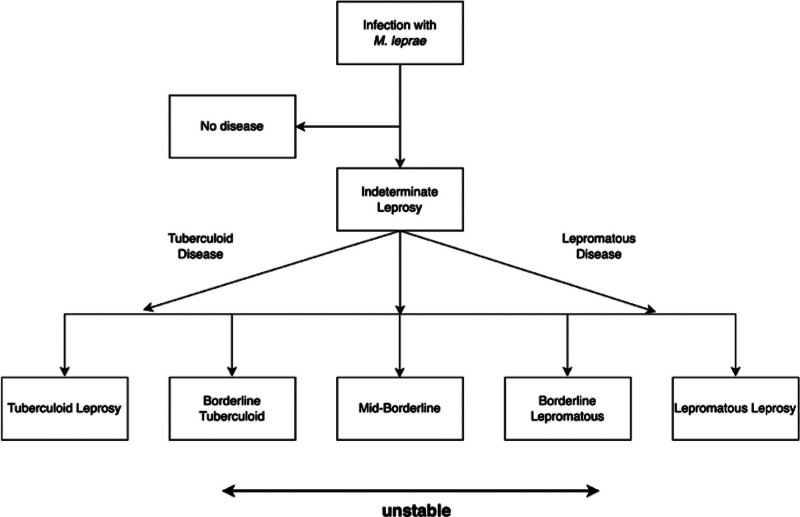
Leprosy classification and clinical spectrum according to Ridley and Jopling.

**Figure 4.**
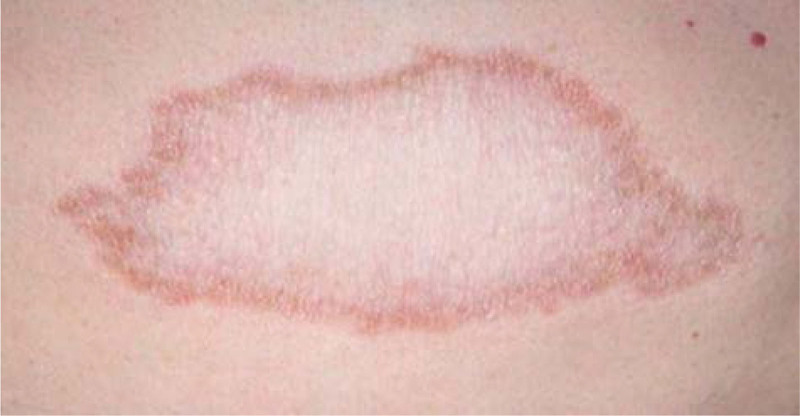
Tuberculoid leprosy: lesion with a single, stable, hairless plaque and well-defined borders (photograph courtesy of Eichelmann K et al).

**Figure 5.**
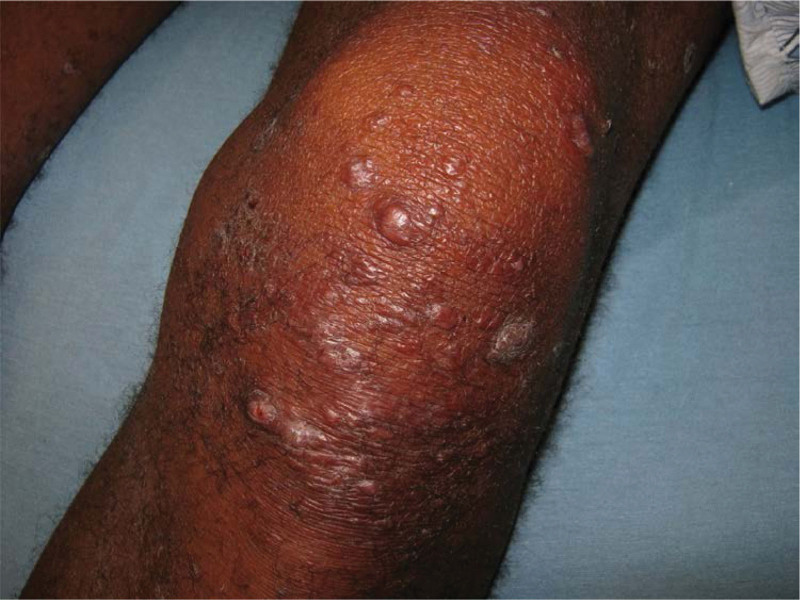
Lepromatous leprosy: lesion with diffuse thickening, numerous discrete, and confluent nodules (photograph courtesy of White C et al).

The clinical manifestations of leprosy vary according to the patient’s immune response to the infection. Several classifications of leprosy have been proposed. The polar system of leprosy was initially defined by Rabello in 1936 and its polarity was described.^[31]^ Since 1953, the Madrid classification, based on clinical aspects, includes tuberculoid, lepromatous, borderline, and indeterminate forms, which were included in the WHO recommendations and were prevalent until 2002.^[32]^ In 1966, Ridley–Jopling introduced a leading classification scheme using a method based on the clinical spectrum, bacteriology, immune status, and histopathological findings of patients with leprosy (Fig. 3).^[33,34]^ Various clinical pictures and histopathological presentations of leprosy are related to host cell-mediated immune (CMI) reactions to *M leprae*, where infected patients fall within this classification model.^[35]^ The Ridley–Jopling classification system categorizes the disease into 2 poles and an intermediate state, which includes polar tuberculoid leprosy (TT) (Fig. 4), borderline tuberculoid leprosy (BT), mid-borderline leprosy (BB), borderline lepromatous leprosy (BL), and lepromatous leprosy (LL) (Fig. 5).^[36,37]^ However, indeterminate leprosy (IL) is commonly present in the sixth category.^[38]^

Immune response polarization is a critical element in the pathogenesis of leprosy and in determining its clinical manifestations. Leprosy cases present a T-cell helper 1 cytokine response and scarce skin lesions, with very few *M leprae classified as having tuberculosis*. In TT cases, macrophages are activated and CD4 T cells remarkably increase. There is a low level of *M leprae*-specific humoral immunity in TT cases; in contrast, cases that present with an impaired Th2 cytokine response and widespread skin lesions of various types, ranging from diffuse skin involvement to nodular infiltrates (called lepromas), significantly decreased in CD4+ T cells, but rather have numerous CD8+ T cells, containing a higher number of *M leprae* bacteria, are classified as lepromatous infections.^[31,32,38]^ The leprosy of borderline forms express immunological dynamics. The borderline group had a mixed histopathological aspect and a progressive reduction in the CMI response from BT with predominantly tuberculoid features to BL forms with predominantly lepromatous features.^[37,38]^ BB was observed between the 2 groups, with a typical punched-out or dome-shaped lesion. The interaction of TH1/Th2 alone cannot fully explain the response to leprosy. Other T cell subsets, such as regulatory T (Treg) and T-cell helper 1 cells, play critical roles in host immunity.^[37–39]^

Indeterminate leprosy, which is an early stage of the disease with one or a few pale skin lesions, has not yet developed a CMI reaction to *M leprae* and can remain indeterminate for a long time or progress to one of the established forms of the disease depending on host immunity.^[39–41]^ This classification has significant concordance with the density of *leprae* bacilli in the dermis. Usually, the bacteriological index (BI) is expressed on a logarithmic scale with scores ranging from 0 to ≥6.^[35,41]^

In 1982, WHO developed a simplified operational classification system in which patients were classified into paucibacillary (PB) and multibacillary (MB) cases:^[42]^ 5 or fewer lesions were classified as PB, and more than 5 lesions were classified as MB. Although this classification is efficient, several reports have revealed that 30% of leprosy patients might be incorrectly classified as PB using this system and might therefore remain undertreated.^[41]^

### 4.1. Leprosy reactions

Leprosy reactions are acute or subacute immune responses to *M leprae.*^[43]^ The skin and nerves are the essential organs involved, observed previously, during the natural history of the disease or even after successful antibiotic treatment.^[44,45]^ Leprosy reactions are categorized clinically and histopathologically as types 1 and 2, respectively.

**Figure 6.**
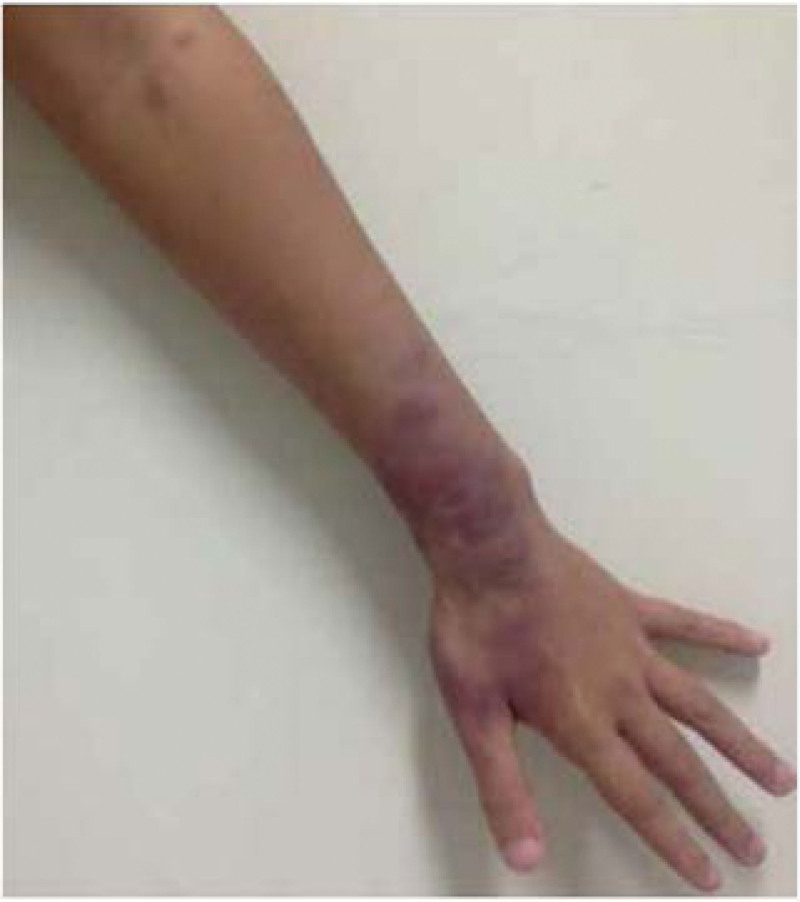
Type 1 reaction: lesions with erythema, swelling, papules, and plaques. (photograph courtesy of Oliveira MB et al).

Pathophysiologically, type 1 reaction (reversal reaction) is a type IV hypersensitivity reaction to *M leprae* antigens (Fig. 6). Previous immunophenotyping studies have shown that the number and proportion of CD4+ T cells are increased in active skin lesions; approximately 30% of patients are classified into the BT, BB, and BL forms.^[31,44]^ This kind of immune response in type 1 reactions can be reversed (reversal, pseudo-exacerbation, or ascending reactions) or worsened (degradation or descending reactions).^[43,45]^ They are the critical reasons for nerve damage in leprosy.^[37]^ The innate and adaptive immune responses are involved in the pathogenesis of type 1 reactions. The most common clinical manifestations are hyperesthesia, urticarial swelling of the leprosy skin, skin ulceration, and sometimes associated with neuritis.^[45,46]^ Most type 1 reactions occur within 12 months of treatment initiation.^[47]^ Upon adequate treatment, borderline patients may upgrade to the TT pole of the clinical spectrum due to reduced bacterial load and increased CMI; however, in untreated patients or upon inadequate treatment, clinical manifestations may downgrade to that of the LL form because of worsened cellular immunity and increased bacterial load.^[46,47]^

**Figure 7.**
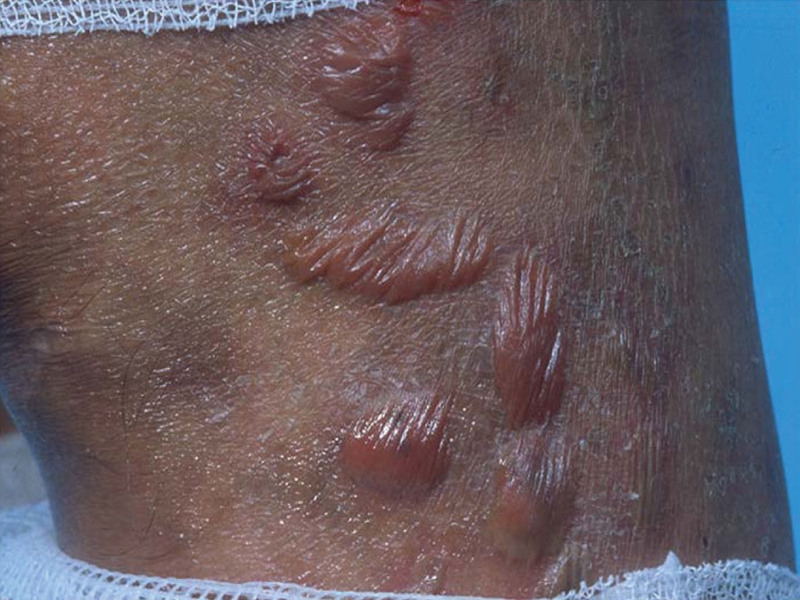
Type 2 reaction: lesions with erythema multiform-like bullous (photograph courtesy of Alemu Belachew W et al).

Type 2 reactions (erythema nodosum leprosum [ENL]) are clinically characterized by painful reddish cutaneous or subcutaneous nodules (Fig. 7), unlike classic erythema nodosum. These have been proposed to be manifestations of type III humoral hypersensitivity reactions.^[48]^ It is found that ENL patients present with high levels of anti-*M leprae*. In ENL lesions, *Leprae* immunoglobulins include IL-6, IL-8, and IL-10 mRNA.^[37]^ Tender erythema skin nodules are not confined to the skin but can involve the entire body with skin ulceration and necrotic lesions.^[48,49]^ They occur mainly on the extensor aspects of the extremities, face, and trunks. Pathophysiologically, ENL is an immune-complex vasculitis that usually occurs in LL and occasionally in patients with BL.^[49,50]^ ENL can be detected before, during, or after treatment initiation; however, it is mainly observed in the first year after treatment.^[50]^ Systemic reactions such as glomerulonephritis, lymphadenitis, iridocyclitis, and orchitis may be accompanied by type 2 reactions.^[49]^ The risk factors included stress, MDT, vaccination, pregnancy, comorbidities, and injuries.^[34]^

Lucio phenomenon is a life-threatening episode that usually occurs in non-treated or inadequately treated diffuse forms of LL, and is always associated with advanced forms of leprosy and a higher bacillary load.^[51]^ It may be a necrotic variant of ENL, although it has certain different features.^[52]^ Clinically, it is frequently an afebrile reaction characterized by extensive erythematous skin lesions that may progress to bullous infiltration, hemorrhagic infarcts, thrombosis, and necrotic erosion; eventually, irregular atrophic scars are left behind.^[53]^ Alopecia, nasal septal perforation, eruptive telangiectasia, hepatosplenomegaly, lymphadenopathy, sepsis, and even death may occur if the disease is not treated immediately.^[54,55]^ Lucio phenomenon is histopathologically present, with many *leprae* bacilli aggregating in the vascular endothelium, lesions with fibroid necrosis, leukocytoclastic vasculitis, and ischemic epidermal necrosis.^[51]^ Although some cases have been reported in Europe and Asia, most have been documented in Central and South America and among immigrants from these areas.^[52]^

### 4.2. Neurological reactions

Neuropathy and related disabilities are the complicated consequences of leprosy. Peripheral neuropathies are a group of disorders that lead to motor or sensory abnormalities in the distribution of the nerves.^[56,57]^ Peripheral neuropathies can be classified as traumatic or nontraumatic neuropathies. Tricking, inflammation, infection, and neoplasm are the most common etiologies of nontraumatic neuropathies. Despite a much better understanding of the theories of *M leprae* invading the peripheral nerves, the detailed mechanisms underlying the pathogenesis of leprosy remain unclear. Although sensory loss is a common feature of leprosy, neuropathic pain has also been observed.^[57,58]^ *M leprae*, which exhibits tropism for Schwann cells in nerve sheaths, invades dermal free nerve endings, damages peripheral nerves, and causes neuritis.^[59]^ Leprosy neuritis differs according to the response to cellular inflammation of the nerves and type of leprosy. In TT, the ulnar and common peroneal nerves are commonly involved; in LL, more peripheral nerves are affected due to a higher bacterial load, and even the facial and trigeminal nerves may be affected.^[57]^

Pure neuritic leprosy (PNL) is a form of leprosy in which only the peripheral nerve is involved, in the absence of skin lesions.^[59]^ PNL commonly affects the ulnar nerve and leads to nerve enlargement and loss of function.^[57,59]^ PNL most frequently occurs in Nepal, Brazil, and India.^[58,60,61]^ A younger age (15–30 years), male sex, and LL form were higher risk groups.^[62,63]^ Effective treatment for leprosy is available, but neuropathy remains a significant problem if the diagnosis and treatment of leprosy are delayed. Because no skin lesions were present, it was difficult to diagnose or delay the diagnosis of leprosy. Clinicians must be vigilant regarding leprosy and related diseases for differentiated diagnosis. In patients with leprosy, MDT and corticosteroids are the primary treatments for neuritis and subclinical neuropathy.^[58]^ Furthermore, postexposure prophylaxis with single-dose rifampin (SDR) in endemic regions and enhanced prophylactic regimens have been implemented in some situations.^[58,59]^ In the future, the study and introduction of new therapeutics for leprosy neuropathy is needed.

## 5. Diagnosis

### 5.1. Clinical findings

Evaluating the clinical manifestations is the first step in diagnosing leprosy, considering the symptoms first, followed by cutaneous signs.^[64,65]^ All the probable cases were examined. Clinical presentations depend more on the host’s CMI reaction against *M leprae* bacilli than on the pathogenesis of the bacillary infection. Skin lesions are typically clinically observed. Suppose that no adequate treatment is provided to patients with *M leprae* infections. In such cases, the disease may permanently spread to the nerves, eyes, and mucosa of the nose, mouth, limbs, or other organs.^[34]^ However, one of the considerable difficulties in diagnosing leprosy is examining it with a list of differential diagnoses of dermatological diseases, particularly in non-endemic countries where leprosy is rare or eradicated.^[40,64]^ A thorough review of personal and family history, including travel to or residence in a country, is essential when diagnosing leprosy.

The WHO recommends making a diagnosis of leprosy if the following criteria are met:^[36]^ (1) pale (hypopigmented) or reddish skin macules with loss of sensation; (2) thickness or enlargement of peripheral nerves with loss of sensation and/or weakness of muscle supplied by the nerve; and (3) presence of acid-fast bacilli (AFB) in a slit skin smear (SSS)/biopsy. When all 3 criteria were met, the accuracy of leprosy diagnosis was as high as 95%.^[34,36]^

Impairment of temperature sensation on the affected skin, thickening of nerves, cranial and limb neuropathies, and trophic changes are common clinical symptoms/signs of leprous neuropathy.^[59]^ Hence, examination of the cranial, peripheral nerves, and anterior chamber of the eye at superficial locations is needed. Skin lesions with hypo/anesthetic sensations in patients with leprosy tend to be located on the face, buttocks, trunk, and ear lobes.^[66–68]^ Loss of pinprick or light-touch sensation helps make a differential diagnosis between leprosy and other dermatological diseases. In some patients, reduced cutaneous sensations and diminished sweating due to the sensory and autonomic nerves were observed.^[65,67]^ Semmes-Weinstein monofilaments are used to assess and monitor tactile sensations in specific areas of the nerve trunks of the hands and feet.^[65]^ Many diseases with dermatological and neurological manifestations should be considered in the differential diagnosis, such as juvenile idiopathic arthritis and hypertrophic lichen planus.^[69]^ Skin biopsy can aid in the diagnosis of leprosy with atypical clinical presentations.^[34,68]^

### 5.2. Bacteriological diagnosis

Table 1 summarizes the laboratory test results for leprosy. Bacilloscopy of the SSS and histopathological examination are the primary methods used to confirm the diagnosis of leprosy. Bacilloscopic examination evaluates the bacillary load and morphology. The slit skin smear exam has a specificity close to 100%; however, it has low sensitivity, since it is positive in only 18% to 30% of infected patients.^[70,71,81]^ A patient with a positive result on the SSS test is classified as having MB; however, a negative result does not rule out a clinical diagnosis of leprosy or PB.^[42,81]^ Previous studies have reported negative results in many patients with PB or primary neuritis.^[33]^ Thus, a polymerase chain reaction (PCR) test increases the sensitivity and specificity of *M leprae* detection, especially in patients with low bacilli load.^[81,82]^ For SSS examination, the most common sites for obtaining specimens are active lesions, long-period persistent lesions with loss of sensation, tissue fluid in the ear lobes, lesions in the elbow, and lesions in body areas with cooler temperatures.^[70]^ After obtaining the specimen, Fite Faraco or modified Ziehl–Neelsen staining was performed for AFB detection. The bacilli were counted and graded using a BI based on the Ridley logarithmic scale, and the percentage of live bacteria was calculated (morphological index).^[38,83]^ For the AFB staining technique, at least 10^4^ bacilli per gram of tissue are required for reliable detection under a microscope.^[83]^

**Table 1:** A summary of sensitivity/specificity of leprosy laboratory tests.

Type of test	Author (year of reported)	Countries/regions	Sample size	Sensitivity/specificity (%)	Reference
Slit skin smear	Banerjee et al (2011)	India	164	Sensitivity: 1.8–59.8 Specificity: 100	^[70]^
Slit skin smear	Lima et al (2022)	Brazil	345	Sensitivity: 24.5 Specificity: 100	^[71]^
PCR	Siwakoti et al (2016)	Nepal	50	Sensitivity: 44–78	^[72]^
qPCR	Azevedon et al (2017)	Brazil	151	Sensitivity: 80.1–84.9	^[73]^
qPCR	Manta et al (2019)	Brazil	HHC: 2437	Sensitivity: 50% Specificity: 94%	^[74]^
PCR	Lima et al (2022)	Brazil	345	Sensitivity: 41 Specificity: 100	^[71]^
PCR	Sevilha-Santos et al (2022)	Brazil	56	Sensitivity: 50–70. Patients with BIs < 2 + had lower sensitivity	^[75]^
qPCR	Sarath et al (2023)	India	32	Sensitivity: 33–100 Specificity: 100	^[76]^
Serological test (anti-PGL-1 IgM)	Leturiondo et al (2019)	Brazil	Healthy:530 Patients: 292	Sensitivity: 81.0 Specificity: 81.7%.	^[77]^
Serological test	Tiemi Nagao-Dias et al (2019)	Brazil	68 (age 4–15 years)	Sensitivity: 25.0% (3.2–65.0%). Specificity: 100.0% (92.1–100.0%)	^[78]^
Serological test (anti-PGL-1 IgM)	Albuquerque et al (2022)	Brazil	466	Sensitivity: 24.1 (95% CI 13.0–38.2)- 76.0% (95% CI 61.8–86.9)	^[79]^
Serological test (anti-Mce1A IgM)	Lima et al (2023)	Brazil	New HD cases: 200 HHC: 105 HEC: 100	Sensitivity: 76.5% (70.0–82.2). Specificity: 88.0% (80.0–93.6)	^[80]^
Serological test (anti-PGL-1 IgM)	Lima et al (2023)	Brazil	New HD: 200 HHC: 105 HEC: 100	Sensitivity: 34.6% (28.5–41,2). Specificity: 96.0% (90.1–98.9)	^[80]^

Anti-PGL-1 = anti-phenolic glycolipid antigen-I isotype; BIs = bacterial indices; HD = Hansen’s disease; HEC = healthy endemic control; HHC = household contact; Mce1A = mammalian cell entry 1A protein.

Histopathological examinations are often performed to confirm clinically suspected cases. Adequate biopsy of the appropriate site is necessary. Additionally, it is used as one of the criteria in the Ridley–Jopling spectral classification and differentiates a leprosy reaction.^[33,84]^ Skin, tissue, and nerve biopsies are essential for diagnosing leprosy. Specimens were collected from the margins of the persistent lesion with loss of sensation or the entire dermis with active skin lesions, and stained using the Fite Faraco or modified Ziehl–Neelsen method.^[83,84]^ Biopsy specimens were evaluated for a granulomatous reaction against *M leprae*, AFB bacterial index of granuloma (BIG), and AFBs in histopathological sections.^[70]^ A nerve specimen taken from a cutaneous or subcutaneous nerve or a more significant nerve was obtained and confirmed by cytology and PCR examinations.^[85–88]^

### 5.3. PCR tests

PCR is a molecular technique that is sensitive for the detection *M leprae* in patients with MB.^[72]^ Diagnosing leprosy through polymerase chain reaction (PCR) has recently become an appropriate technique. The DNA of *M leprae* and *M lepromatosis* was collected from the specimens, and the possible sources of *M leprae* were determined.^[73,76]^ The sensitivity of PCR tests is much lower in cases with negative BI results, which require alternative examinations to confirm the diagnosis.^[74]^ As demonstrated by PCR tests, SSS is less invasive than tissue biopsy. Compared to patients with negative BI or TT results, patients with positive BI or LL results have a higher sensitivity for PCR tests (87–100% vs 30–83%).^[75]^ PCR is a high-cost and labor-intensive technique; thus, it is only used in developed countries to support clinical diagnosis, and is not routinely performed in resource-limited countries.

### 5.4. Serological test

Serological examination could be a straightforward and less invasive technique for estimating *M leprae*.^[81]^ It detects antibody titers and reflects the infection levels of the patient.^[71,81]^ Measuring the antibodies of phenolic glycolipid-1 (PGL-1) against *M leprae* is the broadest method for identifying *M leprae* infection. PGL-1 antibodies against *M leprae* can be measured using enzyme-linked immunosorbent and immunoassays.^[77–80]^ The levels of PGL-1 antigens in clinical specimens are a valuable parameter for predicting bacterial load and can be used to monitor the effectiveness of chemotherapy against leprosy.^[89–91]^ These antigens are present on the cell walls of *M leprae* and can be detected early in patients, thus providing timely treatment.^[91]^ PGL-1 antibody detection is commonly used to classify patients with leprosy into MB and PB groups. At the same time, it is helpful in the early detection of MB but is unsuitable for detecting patients with PB.^[77]^ The sensitivity of the PGL-1 test in patients with MB is approximately 76.8%.^[77]^ Measuring of PGL-1 antibodies also provides epidemiological information on leprosy in a population. Although serological parameters based on the PGL-1 antigen may not be used as a tool in the confirmed diagnosis of leprosy, they may help to follow the treatment effect and actively search for new cases of leprosy.^[79,80]^ Serological tests for patients with *leprae* infection have many limitations in diagnosing *M leprae* infection and in differentiating between contact and infectious cases.^[90]^ Thus, it is necessary to develop a simple and low-cost diagnostic technique to monitor treatment and household transmission in regions with limited resources and endemic countries.

Overall, bacilloscopic SSS is an exclusive laboratory test used to confirm the diagnosis of leprosy. However, a previous study^[81]^ revealed that SSS exhibits low accuracy in diagnosing leprosy owing to its low sensitivity in detecting *M leprae*. In contrast, PCR and serological tests allow for more sensitive and accurate diagnosis of leprosy. Thus, PCR and serological tests have higher sensitivities than SSS for diagnosing *M leprae* infections. To improve the diagnosis of patients with leprosy in the earlier stages of infection, PCR and serological tests are recommended to examine leprosy patients and household contacts.^[92]^

### 5.5. Other diagnostic procedures

#### 5.5.1. Lepromin test

Lepromin test is not primarily used to diagnose leprosy. Alternatively, it indicates the individual immune responses of patients with leprosy. Following intradermal injection of a standardized dose of heat-killed *M. laprae*, a delayed-type hypersensitivity (DTH) reaction develops, in which an early (Fernandez) reaction occurs within 48 hours and a late (Mitsuda) reaction occurs within 3 to 4 weeks at the site of lepromin injection, if the body contains antibodies to *M. Leprae*.^[80,93]^ In the lepromin test, patients with PB may have evoked a strong DTH skin reaction; however, no skin response was observed in patients with MB.^[94]^ However, both positive and negative results can be obtained in healthy individuals. Thus, it is only helpful for classification and prognostic purposes.^[93,94]^ Because it is produced from biological materials, it may cause sensitization. Therefore, many clinicians have opposed its use as a diagnostic tool for leprosy.

#### 5.5.2. Electrophysiological nerve tests

Nerve involvement is observed in all types of leprosy, and occurs without skin lesions. Electrophysiological nerve tests are the main examinations for the diagnosis of peripheral neuropathies.^[66,67]^ In endemic regions, clinicians should be alert to the neurological symptoms of leprosy in endemic regions. Impairment of the sensory nerve function is often the first symptom of leprosy neuropathy. Sensory function abnormalities are typically detected using monofilament or ballpoint tests, while motor function is assessed using voluntary muscle tests. Nerve Conduction Studies (NCS) and Warm Detection Thresholds (WDT) were able to identify patients with leprosy neuropathy earlier.^[58,95]^ The NCS method assesses the large Aβ fibers responsible for vibration, touch, and pressure perception. The WDT method tests small myelinated Aδ- and unmyelinated C-fibers, which mediate pain and warm sensations and govern autonomic function.^[96–98]^ The neuropathies caused by drug therapy sometimes cannot be differentiated from neuropathy caused by leprosy. NCS may play a role in detecting complications associated with leprosy therapy. The sensitivities of NCS and WDT for detecting patients with leprosy neuropathies were 16% and 83%, respectively, whereas their specificities were 88% and 82%, respectively.^[96]^ NCS is not specific for diagnosing leprosy, but it helps predict the extent of neuropathy and monitor therapeutic response. These tests are impractical in resource-limited countries and under field conditions because they require climate-controlled rooms and highly trained staff, and are expensive.

#### 5.5.3. Ultrasonography

An accurate diagnosis of nerve deformities is important for classifying, diagnosing, and treating leprosy.^[99]^ Assessment of peripheral nerve thickening by palpation is subjective and may differ between clinicians. On examination with high-resolution ultrasound, a characterized image of the surrounding epineurium was found.^[100,101]^ The peripheral nerves of patients with leprosy were examined using ultrasonography to diagnose nerve involvement by detecting increased vascularity and nerve thickening.^[102,103]^ A unique sonographic picture of nerve enlargement in patients with ulnar neuropathy due to *M leprae* infection has been noted.^[104,105]^ The enlargement starts at the ulnar sulcus, reaches a maximum of 4 cm above the medial epicondyle, and decreases further along the tract. This characteristic image of focal enlargement of the nerves, particularly at entrapment sites, and epineural thickness can help diagnose the pure neuritic type of leprosy.^[105]^ Ultrasonography can reach clinically nonpalpable sites to evaluate nerve involvement and fascicular architecture alterations.^[103]^ Compared to clinical examination by palpation, high-resolution ultrasound was more sensitive in detecting enlarged nerves (20% vs 47%, *P* < .001).^[99]^ Moreover, it is a low-cost, noninvasive technology.

#### 5.5.4. Magnetic resonance neurography

Magnetic resonance neurography (MRN) is primarily used to evaluate the extent of the proximal nerve involvement.^[102]^ MRN is an objective diagnostic tool for peripheral neuropathy (PN). The nerves affected by leprosy present a specific image of nerve enlargement, increased signal intensity on magnetic resonance imaging, and nodular enhancement.^[102]^ Moreover, subacute and chronic phases of edema, thickening, nerve calcifications, and nerve microabscesses were observed.^[101]^ According to MRN findings, a nerve is classified into group I (as usual), group II (enlargement and fascicular abnormalities), or group III (disappearing fascicular structure).^[106]^ In a study with a total population of 54 patients with MB leprosy, 29 patients were *Mycobacterium leprae* positive in sural nerve biopsies, and 5 patients (5/29; 17.24%) had MRI abnormalities in the central nervous system, spinal root ganglion, and/or brachial plexus.^[107]^ MRN may play a role in the diagnosis of leprosy neuropathy.^[103]^ Besides peripheral nerves, brain and spinal cord involvement has also been determined using MRN.^[108]^

Both the central and peripheral nervous systems can be affected by leprosy; however, peripheral nerves, including the motor, sensory, and autonomic divisions of the peripheral nervous system, are more commonly involved.^[95]^ The characteristics of peripheral nerves in leprosy have been extensively studied; however, it has been postulated that bacilli do not invade the central nervous system owing to their inherent neurotropism for the peripheral nerves.^[109]^ Clinical and histopathological findings of the skin and nerve tissue and PCR results are usually sufficient to diagnose leprosy. With the increased global mobility of patients, physicians should be aware that leprosy mimics other disorders and should be alert when making an early diagnosis.^[109]^ Ultrasonography and MRN are essential for evaluating nerve involvement in the early diagnosis of leprosy/leprosy reactions and for effective patient management, thus preventing nerve damage in patients and subsequent disabilities.^[109,110]^

## 6. Treatment

Dapsone was the first antibiotic to be used for leprosy treatment in the 1950s. However, dapsone-resistant *M leprae* was detected in leprosy controls after almost 30 years of dapsone monotherapy.^[111]^ Dapsone resistance occurs because of specific mutations within the highly conserved p-aminobenzoic acid-binding site of *E coli* dihydropteroate synthase.^[112]^ To overcome the problem of drug-resistance *M leprae* and to improve treatment efficacy, MDT against leprosy, including dapsone, rifampicin, and clofazimine, was suggested by the WHO in 1981.^[36]^ The WHO modified these recommendations several times and standardized them worldwide in 2018 (Table 2).^[36]^ It is reasonable to conclude that the advantages of MDT include the decreased prevalence of dapsone resistance, infectiousness becoming unlikely, and reduced rates of recurrence and reactions.^[34]^ The 3 drugs of first choice for treating leprosy are rifampicin, clofazimine, and dapsone (diamino diphenyl sulfone). The WHO established 2 treatment regimens:^[36]^ Patients with PB are recommended to be treated with rifampicin, dapsone, and clofazimine for 6 months, whereas patients with MB are recommended to be treated with rifampicin, dapsone, and clofazimine for 12 months. Both regimens were administered on an outpatient. In endemic regions, all patients take drugs monthly, under supervision and documentation. Although the prevalence of leprosy has decreased due to the widespread use of MDT globally, rifampicin-resistant *M leprae* (incidence = 11% [95% CI, 7–15%]) remains a concern.^[111]^ Rifampin is recommended as a critical anti-leprosy chemotherapeutic antibiotic; however, rifampin resistance is associated with rpo*β* mutations.^[111]^

**Table 2:** Treatment regimens for leprosy as recommended by the World Health Organization.

Population	Medication	Dose	Duration	
			Paucibacillary leprosy	Multibacillary leprosy
Adults	Rifampicin	600 mg/month	6 months	12 months
	Clofazimine	300 mg/month + 50 mg/day	6 months	12 months
	Dapsone	100 mg/day	6 months	12 months
				
Children (10–14 years)	Rifampicin	450 mg/month	6 months	12 months
	Clofazimine	150 mg/month + 50 mg/day	6 months	12 months
	Dapsone	50 mg/day	6 months	12 months
				
Children (< 10 years old or < 40 kg)	Rifampicin	10 mg/kg/month	6 months	12 months
	Clofazimine	6 mg/kg/month + 1 mg/kg/day	6 months	12 months
	Dapsone	2 mg/kg/daily	6 months	12 months

When patients start MDT and are noted to be resistant to rifampicin alone or in association with quinolones, they restart an entire course of alternative second-line drug therapy despite the clinical outcome of MDT. The WHO recommends ofloxacin, minocycline, and clarithromycin as alternative treatments in cases of intolerance or resistance to one or more rifampicin and ofloxacin (Table 3).^[36]^ Other regimens have been proposed by the United States of America’s National Hansen’s Disease Program, which have more extended therapeutic periods due to fewer restrictions on cost and the exclusion of clofazimine in PB treatment.^[113]^

**Table 3:** The World Health Organization recommends treatment regimens for drug-resistant leprosy.

Resistance type	Drug therapy	
	First 6 months (daily)	Next 18 months (daily)
Rifampicin resistance	Ofloxacin 400 mg* + minocycline 100 mg + clofazimine 50 mg	Ofloxacin 400 mg* OR minocycline 100 mg + clofazimine 50 mg
	Ofloxacin 400 mg* + clarithromycin 500 mg + clofazimine 50 mg	Ofloxacin 400 mg + clofazimine 50 mg
Rifampicin and ofloxacin resistance	Clarithromycin 500 mg + minocycline 100 mg + clofazimine 50 mg	Clarithromycin 500 mg OR minocycline 100 mg + clofazimine 50 mg

* Ofloxacin 400 mg can be replaced with levofloxacin 500 mg or moxifloxacin 400 mg.

Several studies have indicated that bacterial load in patients with leprosy correlates with IgM antibody titers against *M leprae*-specific PGL-1.^[71,81]^ For the proper treatment of leprosy and the success of leprosy control programs, a combination of classification through the number of lesions and a serological test of the lateral flow of *M leprae* has been developed.^[41,85]^

## 7. Prevention

### 7.1. Prophylactic immunization

Prophylactic immunization to prevent leprosy infection and disease progression is a significant public health effort. Several universal active vaccines have been developed, including DNA techniques and Bacillus Calmette-Guérin (BCG), LepVax (a leprosy vaccine), and *M indicus pranii* vaccines.^[114–116]^ Given the similarities in antigenic makeup between *Mycobacterium tuberculosis* and *M leprae*, new TB vaccines have been proposed to cross-protect against leprosy. Therefore, BCG vaccination is the most effective strategy for the prevention of leprosy.^[115,117,118]^ Previous studies have shown that the protection provided by BCG vaccination against *M leprae* infection ranges from 20% to 90%, depending on the prevalence and features of the infectious agent.^[119–121]^ The coverage rate of BCG vaccination seems to be related to the detection rate of leprosy. BCG vaccination was started in 1978 in Paraguay; the targeted population was newborns at birth and all children under 5 years of age if they had not received the vaccine.^[122]^ In this study, the incidence of leprosy in Paraguay decreased from 8.3 detected cases per 100,000 people in 2005 to 5.0 detected cases per 100,000 people in 2016.^[123]^ Another study indicated that leprosy occurrence would be substantially reduced if combined strategies were implemented, including good BCG vaccine coverage, contact tracing, early diagnosis, infection treatment, and/or chemoprophylaxis among household contacts.^[123]^ However, the effects of BCG on leprosy remain controversial.^[117]^ Highly related mycobacterial pathogens can cause tuberculosis, leprosy, *M. tuberculosis*, and *M leprae*. Given the similarities in their antigenic responses, new vaccines against tuberculosis could cross-protect against leprosy.^[115]^

### 7.2. Chemoprophylaxis

Chemoprophylaxis effectively reduces the incidence of leprosy in individuals with a history of close contact with their patients.^[124]^ The effectiveness of dapsone/acedapsone chemoprophylaxis has been investigated in randomized trials. However, this is not preferred because of the occurrence of drug resistance and poor patient compliance owing to the required long-term administration.^[124]^ Rifampicin is considered a chemoprophylactic agent for leprosy owing to its substantial bactericidal effect against *M leprae.*^[125]^ Treating subjects who had contact with patients with a SDR^[126,127]^ or rifapentine (a cyclopentyl ring-substituted rifamycin)^[128]^ has been proven effective in reducing the risk of *M leprae* infection. It is concerned with the risk that SDR would induce rifampicin-resistant tuberculosis; however, a study refutes this concern and suggests the benefits of rifampicin therapy for leprosy strongly outweigh the risks.^[129]^

Additionally, a study conducted in Bangladesh revealed the protective effects of Bacillus Calmette-Guerin (BCG) and rifampicin.^[130]^ Treating contacts using SDR was recommended by the WHO in 2018.^[34]^ However, examining the additive effect of SDR in patients who have received BCG vaccination is difficult because many patients already have leprosy before the administration of SDR.^[121,131]^ Although the WHO recommends a more extended initial rifampicin dose with moxifloxacin or clarithromycin, further studies are required to trace and identify contacts that need protection.^[132]^

## 8. Conclusion

Leprosy remains a major public health concern worldwide. Clinical manifestations of this condition are broad. Physicians should consider a diagnosis of leprosy if patients present with skin lesions associated with loss of sensation in non-endemic regions, primarily when related to potential exposure through travel to endemic areas or close contact with leprosy patients. If a patient is confirmed to have leprosy, prompt treatment and prevention of disability and/or disease spread are essential. In addition to adequate drug therapy, patients with physical disabilities require further rehabilitation and holistic healthcare. Other studies on chemoprophylaxis of contacts, delay in diagnosis, stigma, discrimination, and mental health are needed.

## Author contributions

**Conceptualization:** Chien-Yuan Huang, Kow-Tong Chen.

**Data curation:** Chien-Yuan Huang, Shih-Bin Su.

**Formal analysis:** Chien-Yuan Huang, Shih-Bin Su.

**Investigation:** Shih-Bin Su.

**Methodology:** Kow-Tong Chen.

**Validation:** Kow-Tong Chen.

**Writing – original draft:** Kow-Tong Chen.

**Writing – review & editing:** Kow-Tong Chen.
